# The effect of learning style and general self-efficacy on satisfaction of e-Learning in dental students

**DOI:** 10.1186/s12909-021-02903-5

**Published:** 2021-08-31

**Authors:** Tahereh Baherimoghadam, Shahram Hamedani, Manoosh mehrabi, Navid Naseri, Nooshin Marzban

**Affiliations:** 1 Department of Orthodontics, School of Dentistry, Shiraz Branch, Islamic Azad University, Shiraz, Iran; 2 Oral and Dental Disease Research Center, School of Dentistry, Shiraz University of Medical Sciences, Shiraz, Iran; 3 Department of e-Learning in Medical Sciences, Shiraz University of Medical Sciences, Shiraz, Iran; 4 Shiraz, Iran

**Keywords:** Distance learning, e-Learning, Student satisfaction, General self-efficacy questionnaire, Learning styles

## Abstract

**Introduction:**

The COVID-19 pandemic has had a significant impact on education. e-Learning has been becoming most popular. Satisfaction of the student is one of important goal of e-Learning, therefore factors affecting this satisfaction should be considered extensively. This study aims to evaluate the effect of learning style and General Self-Efficacy (GSE) on satisfaction of e-Learning in dental student.

**Method:**

Electronic questionnaires were sent to 85 fifth and sixth-year students who had passed the face-to-face orthodontics course in the previous semester and were studying online orthodontics at the time of this study. Three questionnaires were used including Soloman and Felder learning styles index, General self-efficacy questionnaire and Satisfaction questionnaire for online education.

**Results:**

The results of the reliability test showed that Cronbach’s alpha index for the self-efficacy and satisfaction questionnaire was 0.836 and 0.96, respectively. The correlation between satisfaction and the dimensions of learning style showed that the active dimension of processing information had a significant relationship with the level of satisfaction. In the understanding dimension, a relatively strong correlation was observed in the Global dimension. Moderate significant relationship between the total score of self-efficacy and the level of satisfaction has been found.

**Conclusion:**

The results of the present study highlight the necessity of more studies regarding defining effective on student satisfaction during e-Learning. GSES and active learning style in the processing dimension and global learning style in the understanding dimension affect students' satisfaction.

## Introduction

A thriving graduation of dental students can be achieved through a good curriculum and effective educational style. The integration of information and communicational technologies, together with active learning methods in the classroom has made insightful changes in the education of dental students in the recent era. These changes are considered as a global phenomenon since they are happening all around the world regardless of cultural influences, or social and economic status of students [1]. Generation Z is referred to those who were born in 1995 or later. These electronic multitaskers are the most electronically dependent generation and are extremely adaptable to new technology [2]. The students of this generation prefer non-traditional teaching methods and desire to experience logic-based approaches and tentative learning style [1, 3]. Concerning the pedagogical aspects, electronic education alters the passive model of teaching (teacher-centred) to the active (student-centred) model [1]. Regardless of time and place variables, electronic learning (e-Learning) improves the teaching process and provides faster availability of knowledge, and better connections between teachers and students [4].

E-Learning can be considered as new paradigm of online learning on information technology [5]; Teachers are more eager to determine how e-learning can result in better outcomes; this can be achieved by analyzing student satisfaction after e-Learning course. [6]

Many various factors might affect how each individual student take an approach toward learning new information, one of these approaches would be learning style [7].

A combination of cognitive, emotional and physiological characteristics might indicate how a student can learn, which is generally defined as learning style [8]. The presence of various learning styles is related to the different ability and individual preference of students to learn [1]. The knowledge of learning styles grants pedagogical approaches and would provide imperative insights for students and teachers regarding either their strengths or their weaknesses in both teaching and learning practice [1, 9]. The impact of learning style on academic performance of students has been previously verified in studies [10–14]. Several learning-style frameworks have been employed in different health science educational disciplines [1, 15]. Felder and Soloman’s Index Learning Style (ILS) tool describes the characteristic preferences and strengths, regarding the ways that students receive and process information [16]. This tool has been previously employed for dental students [1, 17].

Evidence clearly shows that there is a relationship between self-efficacy and learning style [18]. Self-efficacy refers to ‘beliefs in one’s capabilities to organize and execute the courses of action required to produce given attainments’ [19]. Student self-efficacy has emerged as an imperative construct in educational studies over the last forty years. Self-efficacy has been reported to be an important variable in student learning, since it influences students’ motivation and learning process, the psychological paradigm of self-efficacy has an imperative role in current educational psychology [18]. The evidence shows the direct and indirect impact of students’ self-efficacy on their achievements and that self-efficacy has a predicting and mediating role regarding students’ achievements, motivation and learning [18, 20, 21]. It is reported that pedagogical practices compose a significant influence on student’s precepts of efficacy. It is also postulated that a general cognitive engagement of learning has been strongly associated with self-efficacy perception [22]. Self-efficacy can be measured by an administered tool, a reliable and valid self-report questionnaire, namely Generalized Self-Efficacy Scale (GSES) created by Schwarzer and Jerusalem [23]. Due to COVID-19 pandemic, the policy of physical distancing has been implemented in most of university including dental school [24], and all efforts are made to make e-Learning education as good as possible.

### Research purpose and hypotheses

Until now, few studies evaluated the effect of different factors on student satisfaction; though none of them evaluated their effect on satisfaction with e-Learning. Hence, this study was conducted to scrutinize the influence of self-efficacy and learning style on satisfaction perception of dental students recruited in an orthodontic e-Learning course.

The null hypothesis for the current study was proposed as there are no differences in various learning styles in satisfaction of students with e-Learning. Moreover,the alternative hypothesis was considered as individuals with high GSES will be more satisfied with e-Learning.

## Method and Materials

**Fig. 1:**
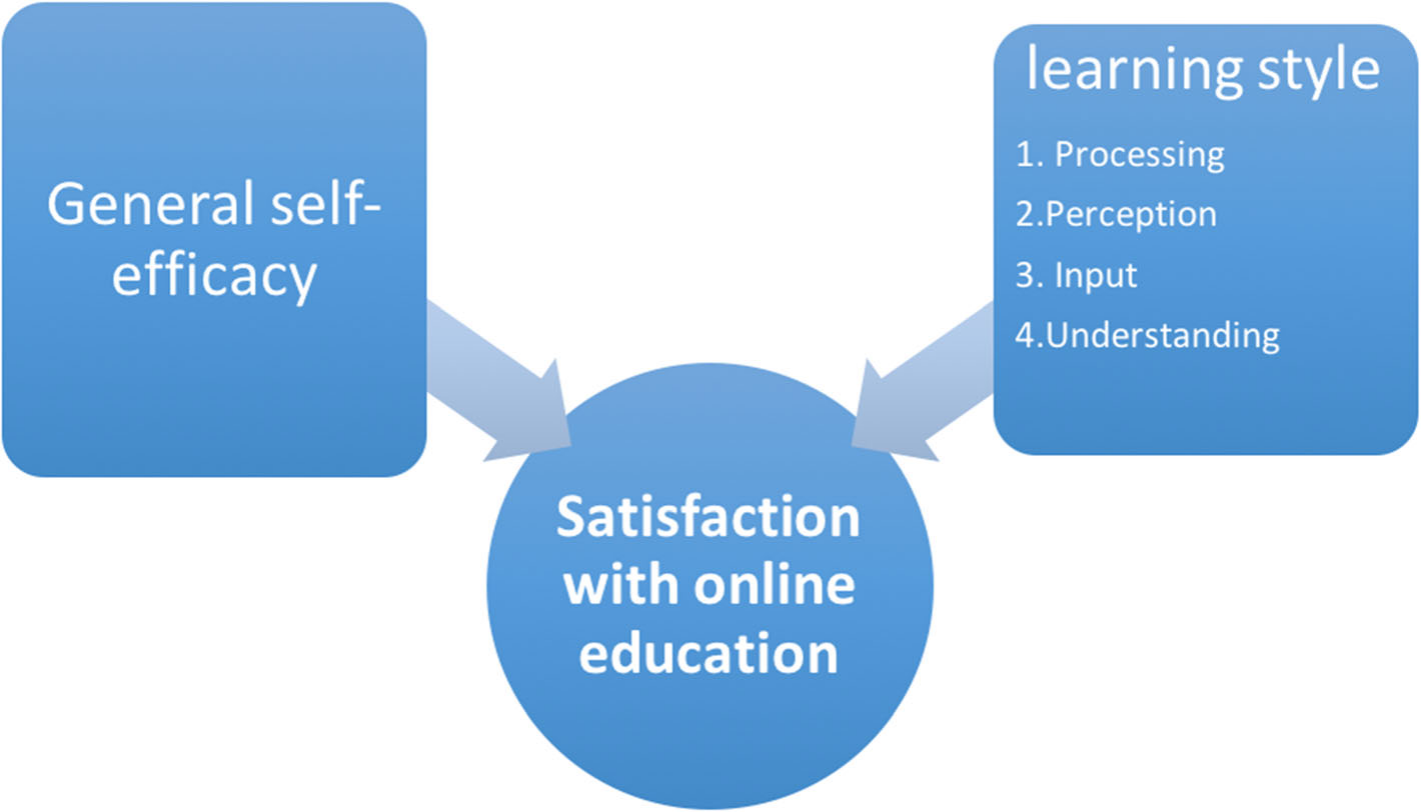
Proposed model

 The Research Council of the School of Dentistry of Shiraz Islamic Azad University has approved the current study. In order to maximize participation and sufficient time to respond during corona pandemic, an electronic questionnaire was developed so that participants could easily complete the questionnaire wherever they were comfortable. The electronic questionnaire was launched on the website for a certain period of time. A total of 85 fifth and sixth-year students who had passed the face-to-face orthodontics course in the previous semester and were studying online orthodontics at the time of this study and were willing to participate in this research were recruited. They were asked to answer the questions of the questionnaire and express their satisfaction regarding their ongoing orthodontics e-Learning course. Before completing the questionnaire, the necessary descriptions were given to the students. At the end of the data collection period, the website portal was closed automatically. In this study, three questionnaires were used including (1) Soloman and Felder learning styles index, (2) General self-efficacy questionnaire, and (3) Satisfaction questionnaire for online education (Fig. 1)

.

### Soloman and Felder learning styles index (SFLSI)

This questionnaire has been approved as a suitable tool for assessing students’ learning styles [14]. The Persian version of this questionnaire, whose validity and reliability has been verified, was used in the study [25]. This questionnaire consists of 44 two-choice questions; it is designed to assess preferences related to four dimensions of learning style. The SFLSI consists of four scales, each with eleven questions. The tool is summarized into the following four scales: (1) Processing Information: active (Learn by trying and enjoying working in groups) or reflective (learning by thinking, preferring to work alone, or with a familiar partner); (2) Perceiving Information: sensing (solid thinker, practical, the desire to learn facts) or intuitive (abstract thinker, learning by discovering relationships, creativity and innovation); (3) Receiving Information: visual (they learn better what they see, visual preference such as pictures, diagrams, and flow charts) or verbal (they learn better what they hear and prefer written and spoken rationalization), and (4) Understanding Information: sequential (The learner tends to understand the content in regular and linear stages and in dealing with complex problems tries to go through the steps logically step by step so that they can solve their problems) or *global* (The learner is holistic and in dealing with complex problems, they first try to understand them and then solve them [1]. After completing the questionnaire, the results were organized in such a way that the respondent learning style priority was classified using all four groups of learning styles. For instance, a score of 1 to 3 indicated that learning in two dimensions was relatively balanced. Results between 5 and 7 indicated a moderate priority for one dimension of the scale and indicate that the student may learn more easily in this dimension. If the student scores were 9 to 11, it indicated that the student had a serious priority over one dimension of the scale, and as a result, if this preference is not supported, the student may face learning difficulties.

### General Self Efficacy Scale

Schwarzer and Jerusalem designed GSES questionnaire, which measures a person’s confidence in their ability to succeed in a variety of situations; it shows a valid relationship between a person’s level of behavioral health and the configuration of health-related habits. This questionnaire consists of 10 items that are scored in a 4-point Likert scale [23]. The validity and reliability of the Persian version of this questionnaire has also been assessed [26]. The answer is that for each phrase, they choose one of the four available options that indicate how similar they are to the phrase. Each of the options is equivalent to a certain score as follows: (1) not at all true = score 1, (2) hardly true = score 2, (3) moderately true = score 3, and (4) exactly true = score 4. This is a one-component questionnaire and the yielded points are added together. The overall score is 10 to 40, which means that the higher the score, the higher the self-efficacy.

### Satisfaction questionnaire

A 3-item satisfaction subscale questionnaire, adapted from Aretino was used to weigh up students’ overall satisfaction with the self-directed, online course [27]. The results of the reliability test for Persian version of satisfaction questionnaire showed that Cronbach’s alpha index was 0.96. Sample items include, I anticipate for more online courses in the future, this online course provided my requirements as a learner, and overall, I was pleased with my online learning experience. The answers to the questions vary from completely dissatisfied (0) to completely satisfied. A higher score indicated a high level of satisfaction.

### Statistical Analysis

SPSS statistical software version 23 was used for data analysis. The sample size was determined to be 85 using the Cochran’s formula and a maximum error of three units. Data sampling method was targeted method. To assess data distribution, Kolmogorov-Smirnov test was employed. The results of the normality test showed that only the self-efficacy variables (total score) and satisfaction rate were normal (p-value > 0.05) and the self-efficacy questions and learning dimensions were not normal individually (p-value < 0.05). Therefore, Pearson correlation coefficient was used to calculate the correlation coefficient of self-efficacy and satisfaction, and Spearman correlation coefficient was employed for learning dimensions and satisfaction. Regression analysis was used to determine the effect of each independent variable on student satisfaction. 20 randomly selected subjects filled out the questionnaire for the second time after a two-week interval to evaluate Test-retest reliability.

## Result

Demographic results showed that the majority of participants (58.8 %) were female students. The mean age of female and male students was (24.11 ± 3.25) and (25.11 ± 3.47), respectively.

**Fig. 2:**
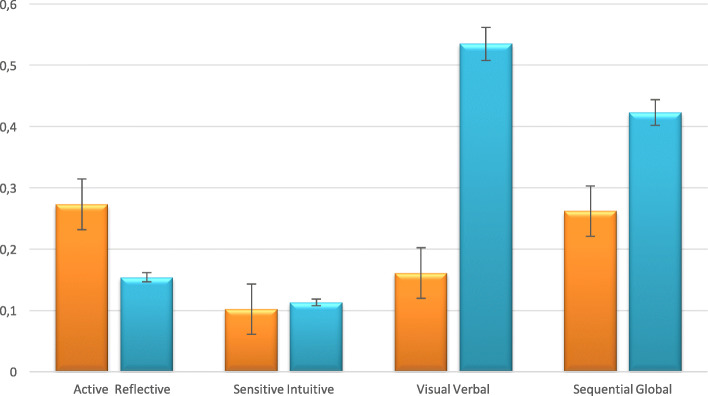
Comparison of correlation coefficients of satisfaction with dimensions of the learning style

The correlation between the questions of satisfaction with education and the dimensions of learning style showed that the active dimension of processing information had a significant relationship with the level of satisfaction. In the understanding dimension, a relatively strong correlation was observed in the Global dimension. (Table 1) A comparison of correlation coefficients of satisfaction with dimensions of the learning styles is shown in Fig. 2.

**Table 1:** Correlation between level of satisfaction with online education and learning style

Dimension of learning style		Number	Spearman Correlation	*P* value
Processing	Active	40	**-0.273**	0.041
	Reflective	45	-0.154	0.123
Perception	Sensing	61	-0.102	0.372
	Intuitive	24	0.113	0.876
Input	Visual	72	0.161	0.335
	Verbal	13	-0.535	0.110
Understanding	Sequential	54	-0.262	0.347
	Global	31	0.423^*^	0.031

The correlation between the questions and the total score of GSE questionnaire and the level of satisfaction with online education has shown a moderate significant relationship between the total score of self-efficacy and the level of satisfaction. (Table 2) The result of regression analysis showed that regression model was non-significant; however, total score of GSE and understanding dimension of learning style can significantly predict the level of satisfaction. (Table 3) The test-retest correlation coefficient was 0.812.

**Table 2:** Correlation between level of satisfaction with online education and general self-efficacy

	q1		q2		q3		q4		q5		q6		q7		q8		q9		q10	Total score
Number	85	**85**		**85**		**85**		**85**		**85**		**85**		**85**		**85**		**85**		**85**
Spearman Correlation	0.295	0.233		-0.06		0.178		0.149		0.285		0.228		0.256		0.137		0.014		0.293^*^
P value	0.059	0.182		0.645		0.443		0.227		0.068		0.142		0.213		0.137		0.432		0.032*

**Table 3:** Regression analysis to determine the effect of general self-efficacy and learning style on the level of satisfaction with online education

	Unstandardized Coefficients		Standardized Coefficients	t	Sig.	R Square	Sig.
	**B**	**Std. Error**	**Beta**				
**(Constant)**	5.733	5.145	0.272	1.114		0.336b	0.132
Processing	-0.109	-0.341	0.751	-0.320	-0.153		
Perception	-0.257	0.293	0.387	-0.874	-0.136		
Input	0.171	0.238	0.476	0.720	0.112		
Understanding	0.182	0.156	0.023	-0.510	**0.085**		
General self-efficacy	0.312	0.166	0.018	1.880	**0.010**		

## Discussion

The results of the present study showed that there is a direct relationship between individual self-efficacy and satisfaction with online education. Considering the relationship between different learning styles and the level of satisfaction with online education, it has been shown that the active learning style in the information-processing dimension showed a significant inverse relationship with the level of satisfaction with online education. However, the global learning style in understanding information dimension has a significant positive relationship with the level of satisfaction with online education. The results of the reliability test of this study were calculated based on Cronbach’s alpha index and the result for the Persian version of the self-efficacy questionnaire was 0.836 and for the Persian version of the level of satisfaction with online education was 0.96. Hence, the questionnaires employed in this study had high reliability. Various factors influence the level of students ‘satisfaction, including the teacher’s popularity and students’ satisfaction with the teacher’s performance, particularly the availability and response rate [25].

In this study, in order to minimize individual differences and errors in practice, the evaluations were tried to be assessed based on the material presented by one lecturer (Orthodontics). In addition, this lecturer has been selected as the top professor (mentor of choice) by students for the past two years and had enough information and complete mastery to online education. In psychology and psychiatry, self-efficacy is defined as a person’s beliefs about the ability to cope with different situations, and its purpose is to assess the individual’s abilities in order to successfully perform a set of measures necessary to achieve the goal. A few studies have been conducted on the correlation between general self-efficacy and students’ satisfaction with online education [26–29].

Many questions have remained unreciprocated, including the relationship between the level of general self-efficacy and the level of satisfaction with the course of study. Bandura points out that the individual self-efficacy is a key factor in the result of any behaviour that the individual decides to engage, therefore, it is necessary to establish a relationship between self-efficacy and performance [16]. In the current study, a significant relationship was observed between the level of general self-efficacy and the level of satisfaction with online education. However, the level of satisfaction with online education was not significantly related to any of the questions of self-efficacy questionnaire alone.

Previous studies have shown that there was a direct relationship between individual self-efficacy and satisfaction with online education [26–29]. The literature review performed by Alqurashi on self-efficacy in online environments has reported mixed results, some of which have observed a positive relationship between self-efficacy and student satisfaction, and some of which have not yielded a relationship between the two variables; Alqurashi attributed these differences in the results to a lack of research studies performed in this field, which make the results not be conclusive [30].

It seems that in addition to the differences in the design of these two studies, another reason for this difference is the scrutiny of the self-efficacy variable in the two studies. In the present study, the self-efficacy has been studied as a general variable, while in some studies this variable has been investigated in three categories: self-efficacy of computer skills, self-efficacy of searching information and the Internet, and self-efficacy of skills with learning management system. Except in the category of self-efficacy of information search skills and the Internet, the other two categories can be used as predictors of student satisfaction with online education [30].

Students have their own unique learning style; however, it may be different in different situations. Learners who prefer different learning styles have different motivations for learning and they also differ in confidence and reading speed. According to Vaishnav and Chirayu, learning style is a set of factors, attitudes and behaviors that facilitate students’ learning in a particular situation; this is the ability of learners to understand and process information [31].

Learning styles affect how students learn and are also influenced by personal experiences, culture, maturity and development. Each learner has distinct and consistent preferred methods of organizing, perceiving, and learning [32]. It has been shown that among dental students, 63 % preferred sensing learning and 42 % preferred visual learning style. Most students were well-balanced between active-reflective (60 %) and global-sequential (68 %) learning dimensions [33]. Similar studies have shown that a large proportion of orthodontic residents prefer sensing, active, and visual learning styles. In addition, they were well balanced in the dimensions of active-reflective and global-sequential learning dimensions [34]. In the present study, dental students were well-balanced in active-reflective learning dimensions.

The results of the present study showed that active learning style in the processing dimension and global learning style in the understanding dimension affect students’ satisfaction with the online course. Cox and Tsai reported that students ‘learning preferences in the sensing-intuitive dimension are strong predictors of students’ learning satisfaction from face-to-face courses [35].

On the other hand, Wang showed that learning styles do not affect participants’ satisfaction with the teaching approach [32]. The results of many studies show a weak relationship between learning styles and learning outcomes [36–38].

In addition to study design, one reason for the difference in these results could be due to the use of various learning style assessment tools in the studies mentioned; obviously, different tools lead to different results. Cheng and Chau (2014) observed the use of the following tools in various studies: the Kolb’s model (1984), the Felder- Silverman (1988) learning style model, the Herrmann brain dominance instrument (1989), the Myers-Briggs (1993) personality type indicator, and Dunn and Dunn model (2003).

However, the authors indicate that the Felder -Silverman model is more appropriate tool than other tools for two reasons. The first reason is that each aspect of the tool is two-dimensional and the second reason is that it is more flexible considering the students’ learning needs in online and blended environments [39].

## Conclusions

The use of technology in education has become widespread and by benefiting from its strength, university teachers have the opportunity to design their own education in a way that leads to deeper and more appropriate learning for students. One of the most important components of e-Learning is the use of a learning management system on which it is necessary to provide the desired training and employing all the tools in it based on learning objectives and in a wise way.

In addition, since we know there are different learning styles in students; appropriate learning activities should be designed based on information and communication technology and presented by using this system. Therefore, students can find their appropriate content based on their needs and learning style, which subsequently brings about their satisfaction with learning and ultimately leads to their academic success. The result of this study opens further doors for future research. Given the situation produced after the COVID-19 pandemic, more and broader studies on online course satisfaction will be useful for students and educators.

## Data Availability

The datasets used and/or analyzed during the present study are available from the corresponding author upon request.
